# Identification of TNFAIP6 as a hub gene associated with the progression of glioblastoma by weighted gene co‐expression network analysis

**DOI:** 10.1049/syb2.12046

**Published:** 2022-06-29

**Authors:** Dongdong Lin, Wei Li, Nu Zhang, Ming Cai

**Affiliations:** ^1^ Department of Neurosurgery The Second Affiliated Hospital‐Yuying Children's Hospital of Wenzhou Medical University Wenzhou Zhejiang China; ^2^ The Second School of Medicine Wenzhou Medical University Wenzhou Zhejiang China

## Abstract

This study aims to discover the genetic modules that distinguish glioblastoma multiforme (GBM) from low‐grade glioma (LGG) and identify hub genes. A co‐expression network is constructed using the expression profiles of 28 GBM and LGG patients from the Gene Expression Omnibus database. The authors performed gene ontology (GO) and Kyoto encyclopaedia of genes and genomes (KEGG) analysis on these genes. The maximal clique centrality method was used to identify hub genes. Online tools were employed to confirm the link between hub gene expression and overall patient survival rate. The top 5000 genes with major variance were classified into 18 co‐expression gene modules. GO analysis indicated that abnormal changes in ‘cell migration’ and ‘collagen metabolic process’ were involved in the development of GBM. KEGG analysis suggested that ‘focal adhesion’ and ‘p53 signalling pathway’ regulate the tumour progression. TNFAIP6 was identified as a hub gene, and the expression of TNFAIP6 was increased with the elevation of pathological grade. Survival analysis indicated that the higher the expression of TNFAIP6, the shorter the survival time of patients. The authors identified TNFAIP6 as the hub gene in the progression of GBM, and its high expression indicates the poor prognosis of the patients.

## 1 INTRODUCTION

Glioblastoma multiforme (GBM), the most common and highly low‐survival primary intracranial tumour, accounts for 14.6% of all brain and other central nervous system (CNS) tumours and 48.3% of malignant tumours [1]. According to CBTRUS report, glioblastoma had the highest average annual age‐adjusted incidence rates in malignant brain tumours, reaching 3.22 per 100,000 population. Its 5‐year relative survival was only 6.8% [1]. Glioblastoma presents highly aggressive, proliferative, and other highly malignant biological behaviours that severely limit the survival of cancer patients. The current standard treatment for newly diagnosed glioblastoma is STUPP plan [2], implying that tumour is surgically excised to the greatest extent possible, followed by concurrent chemoradiotherapy and then maintained with temozolomide chemotherapy. Even with the most rigorous treatment, the average median survival time for glioblastoma patients is just approximately 15 months [3]. Although numerous studies have been conducted to investigate new therapies for glioblastoma, alternative treatment strategies such as targeted therapy [4, 5] and immunotherapy, among others, have not demonstrated substantial effects except in tumour‐treating fields [6, 7].

Previous research has revealed a plethora of glioblastoma genetic and epigenetic aberrations, including mutations in IDH1/2, EGFR, PDGFRA, and NF1, methylation alterations in MGMT promoter, hTERT activation mutations, and so on [8, 9, 10, 11, 12]. These biomarkers are essential for improving glioblastoma categorisation, increasing diagnostic accuracy, predicting patient prognosis, and designing effective molecular targeted treatment medicines. However, current targeted treatment techniques for glioblastoma have made little progress [13, 14]. Researchers believed that the efficacy of single‐drug targeted therapy was limited and that combining multiple targeted therapy medications could benefit patients with glioblastoma. As a result, more research is urgently required to explore new therapeutic targets for glioblastoma and design new therapeutic techniques. Due to the rapid development of bioinformatics, we can conduct an in‐depth analysis of publicly available data. Weighted gene co‐expression network analysis (WGCNA) [15] is an advanced bioinformatics method that constructs highly synergistically changing gene groups based on thousands of genes with the most remarkable expression variances. Then, these genetic modules are correlated with clinical phenotypes to identify essential regulatory genes. WGCNA has been employed in a range of disease models, including tumours [16], neurodegenerative diseases [17], dermatology [18], and mental illness [19], among others. Its findings can also be corroborated by biological investigations or clinical analysis. This study performed co‐expression analysis on expression profiles from samples of 28 patients with glioblastoma and nine patients with low‐grade glioma (LGG). Then, we screened clinical‐related gene modules and performed functional enrichment analysis. Next, we constructed a regulatory network for genes within the module and mine hub genes. Finally, the other two databases were used to verify the expression of the hub gene and its relationship with the prognosis of glioblastoma patients. We anticipate that this research study will assist in explaining the progression of glioblastoma and identify a potential therapeutic target for this cancer.

## 2 MATERIALS AND METHODS

### 2.1 Data of GBM and LGG patients in the study

**FIGURE 1:**
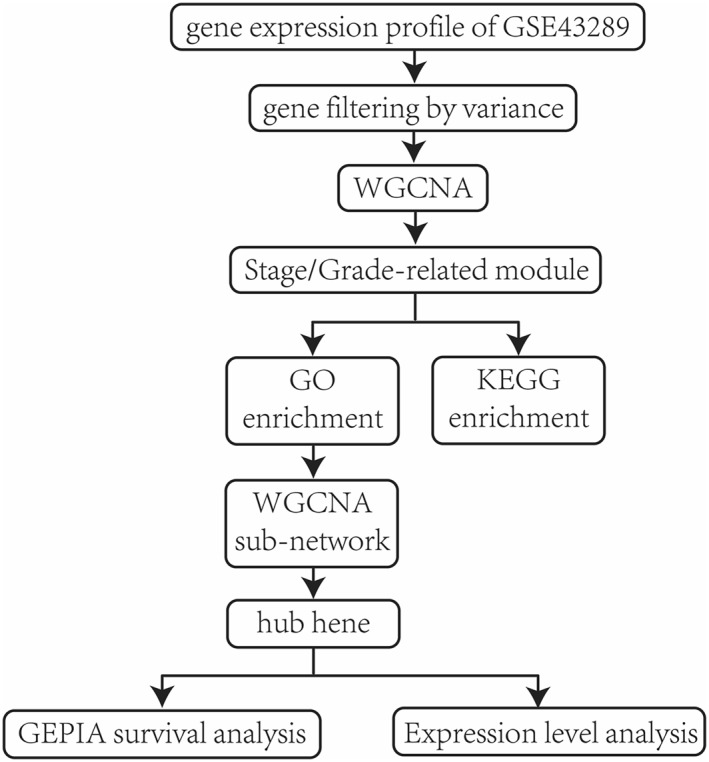
Flow chart of the whole design of this study: data processing, analyses, identification and validation of hub gene

The overall design of this investigation was exhibited in the flow chart (Figure 1). The gene expression profile of GSE43289, including 28 GBM patients and nine samples of LGG patients, was obtained and downloaded from Gene Expression Omnibus (GEO) database (https://www.ncbi.nlm.nih.gov/geo/). The dataset was based on the GPL570 platform of [HG‐U133_Plus_2] Affymetrix Human Genome U133 Plus 2.0 Array.

### 2.2 Data processing

For the pre‐processing of raw data, probe annotation, gene filtering, and outlier sample exclusion were performed. We employed a Perl environment to conduct probe annotation with a microarray platform file. Probes that matched more than one gene were discarded. Whereas, for genes that matched several probes, the average value of these probes was determined as the expression level of these genes. We separated non‐variant genes in the whole gene expression profile based on variance because non‐variant genes were regarded as background noise that influenced WGCNA analysis. Then, using the hierarchical clustering method, we identified and eliminated outlier samples. Gene set enrichment analysis (GSEA) was performed for enrichment analysis.

### 2.3 Enrichment analyses by gene set enrichment analysis (GSEA)

To anticipate gene functions, we used GSEA version 4.1.0 software downloaded from the GSEA website MSigDB database (http://software.broadinstitute.org/gsea/msigdb) to clarify the role of genes in the pathogenesis of GBM. In brief, we used gene ontology (GO) biological process ontology and Kyoto encyclopaedia of genes and genomes (KEGG) gene sets to run GSEA on GBM and LGG samples. The default weighted enrichment method was applied for enrichment analysis. The random combination was set 1000 times. The false discovery rate was added as a correction for Type I errors, and FDRs less than 0.05, together with *p*‐values less than 0.05, were considered significant.

### 2.4 Construction of weighted gene co‐expression network and division of gene modules

We utilised the WGCNA package [20] to construct a weighted gene co‐expression network and division of gene modules under the R environment. First, we adopted Pearson's analysis to correlate the whole genes and created a correlation matrix. Second, we converted the correlation matrix into an adjacency matrix using a suitable soft‐thresholding value β. Considering that the higher the scale‐free fit index is, the better the coincidence with the scale‐free network is, and the higher the mean connectivity is, the better the connection of the whole network is, we calculated the scale‐free fit index and mean connectivity of each β value from 1 to 15, respectively. We chose the β value with the highest mean connectivity when the scale‐free fit index was 0.85. Then, in the sight of indirect correlation between genes, we transformed the adjacency matrix into a topological overlap matrix (TOM). Finally, we divided clustered genes into different modules using hierarchical clustering based on TOM‐dependent dissimilarity metric. We used module eigengenes (the first principal component of a given module) to merge extremely comparable modules (with Pearson's correlation greater than 0.75) into the same module.

### 2.5 Identification of the most clinical‐related modules

The clinical traits of our samples included non‐GBM and GBM, and we calculated the correlation between the clinical traits and module. In brief, the non‐GBM group was refined as 0, and GBM group was refined as 1. Then, we used Spearman correlation analysis between clinical traits and module and calculated the correlation coefficient. The greater the correlation coefficient was, the closer the module was to clinical traits. Moreover, genes in modules of positive correlation with GBM were considered to play essential roles in tumourigenesis. On the other hand, genes in modules of positive correlation with non‐GBM were crucial to maintaining normal biological functions. Hence, we extracted gene modules with the highest correlations with GBM and non‐GBM in subsequent analysis in our study.

In addition, we introduce the conception of gene significance (GS) and module membership (MM). GS was referred to as the association between the gene in the module and the clinical trait. The closer the GS value was to 1, the closer the gene was to the designated clinical traits. In contrast, if the GS value was close to 0, we considered the genes in the module were nearly not relevant to the clinical traits. Module membership was the result of the correlation analysis between genes in the module and eigengenes. Similarly, the higher the MM value was, the stronger the correlation between genes and module. Thus, genes with a high correlation of GS and MM were necessary for being a hub module.

### 2.6 GO and KEGG pathway enrichment analyses

To investigate the specific function of genes in the clinical‐related module, we conducted GO [21] and KEGG [22] enrichment analyses. Gene ontology and KEGG analyses aimed to clarify the terms a certain gene set was enriched in and predict the function of these genes in tumourigenesis. In brief, GO analysis was carried out based on the genes in the module most related to the clinical trait. We used cluster profile R packages and org.Hs.eg.db R packages to conduct GO and KEGG analyses, and the significance was decided by Fisher's exact test. We identified the terms based on the cut‐off of *p*‐value < 0.01 and Benjamin–Hochberg adjusted *p*‐value < 0.05 as significant terms. Then, the top 10 significant terms were visualised using ggplot2, Cairo, and GOplot R packages [23].

### 2.7 Identification of hub genes

Hub gene was highly associated with the whole module and GBM‐trait. We extracted the whole module with the highest connectivity and significance to carry out GO and KEGG analysis. Then, we selected the top GO term based on the significance associated with the development of GBM to construct sub‐network. Cytoscape and its plug‐in and cytoHubba were utilised to find the hub gene from the sub‐network [24]. Based on the maximal clique centrality (MCC) value of each gene, we found several candidate hub genes. Combining with the GS value and MM value of selected genes, the hub gene was finally determined.

### 2.8 Validation of hub genes

To testify the expression level of hub genes in GBM and LGG samples and explore whether the expression level would change with disease progression, we utilised an online gene expression analysis tool known as Gene Expression Profiling Interactive Analysis (GEPIA, http://gepia.cancer‐pku.cn/). Besides, we calculated the hub gene expression in our microarray data. Furthermore, to assess the function of the hub gene in GBM and LGG patients, we visited GEPIA and UALCAN (http://ualcan.path.uab.edu/) to verify the association between the expression of hub genes and the overall survival rate of GBM and LGG patients. Moreover, we used the Human Protein Atlas (HPA) (http://www.proteinatlas.org/) to validate the hub gene expression on the protein level in GBM and LGG samples.

## 3 RESULTS

### 3.1 Data processing

**FIGURE 2:**
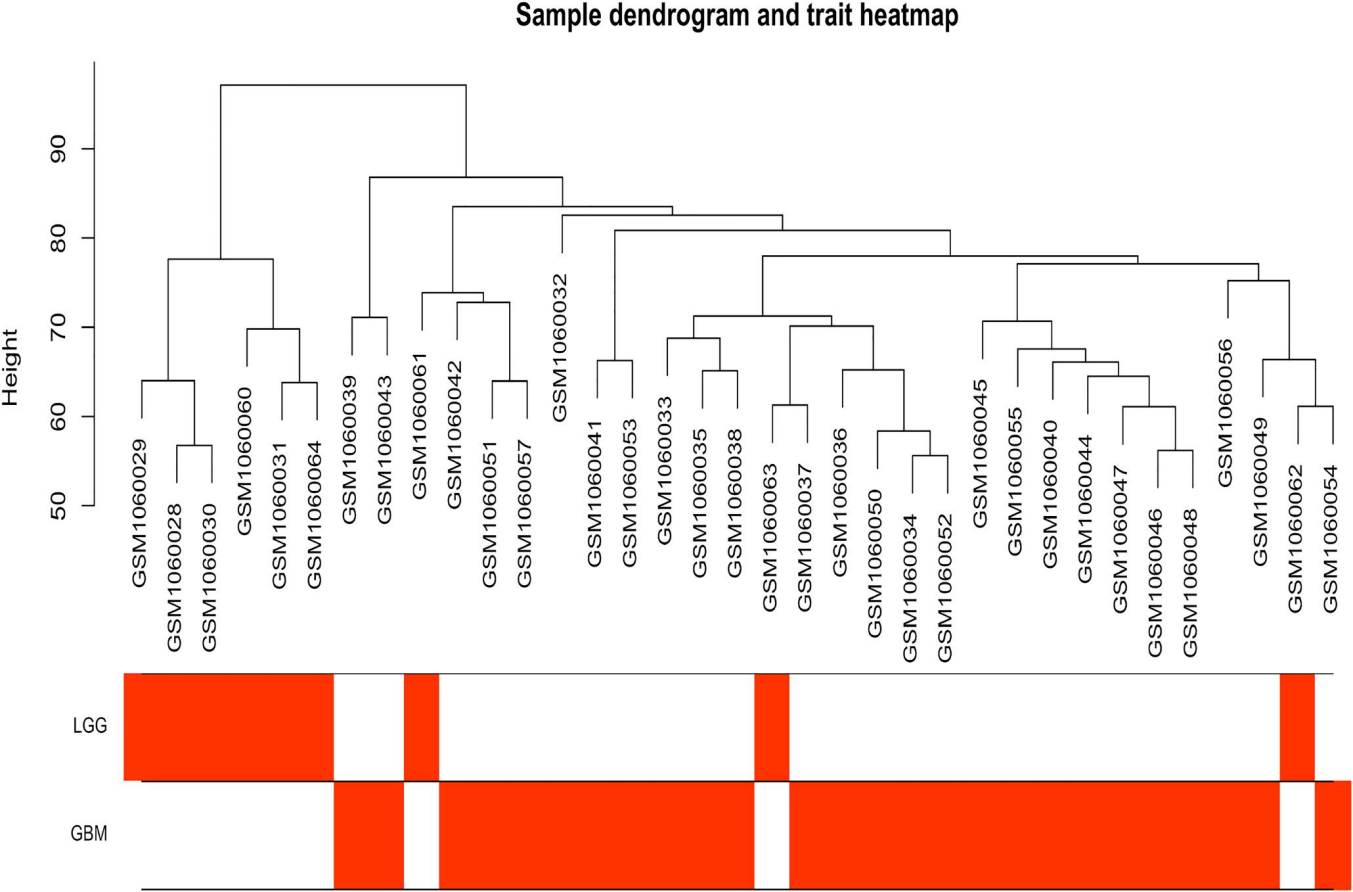
Sample clustering dendrogram and clinical trait heatmap of all the glioma patient samples. The clinical trait of the samples was exhibited with the colour intensity

We obtained a whole expression profile of 23,319 genes after probe annotation and a final expression profile of 5000 genes after gene filtering. After eliminating outlier samples, our selected samples were clustered in sample trees (Figure 2), which exhibited basic clinical traits of each sample. GEO ID and traits of each sample are included in Table S1.

### 3.2 Enrichment analyses by GSEA

We performed GSEA on GO and KEGG gene sets to investigate the function of genes in the development of GBM. The findings revealed that the top five significant GO pathways were ‘syncytium formation,’ ‘myoblast fusion,’ ‘positive regulation of regulatory T cell differentiation,’ ‘pyrimidine nucleoside monophosphate metabolic process’ and ‘integrin‐mediated signalling pathway’ (Figure S1A‐E). The top five KEGG pathways were identified: ‘drug metabolism other enzymes,’ ‘starch and sucrose metabolism,’ ‘pantothenate and COA biosynthesis,’ ‘ECM receptor interaction,’ and ‘focal adhesion’ (Figure S1F‐J).

### 3.3 Construction of weighted gene co‐expression network and division of gene modules

**FIGURE 3:**
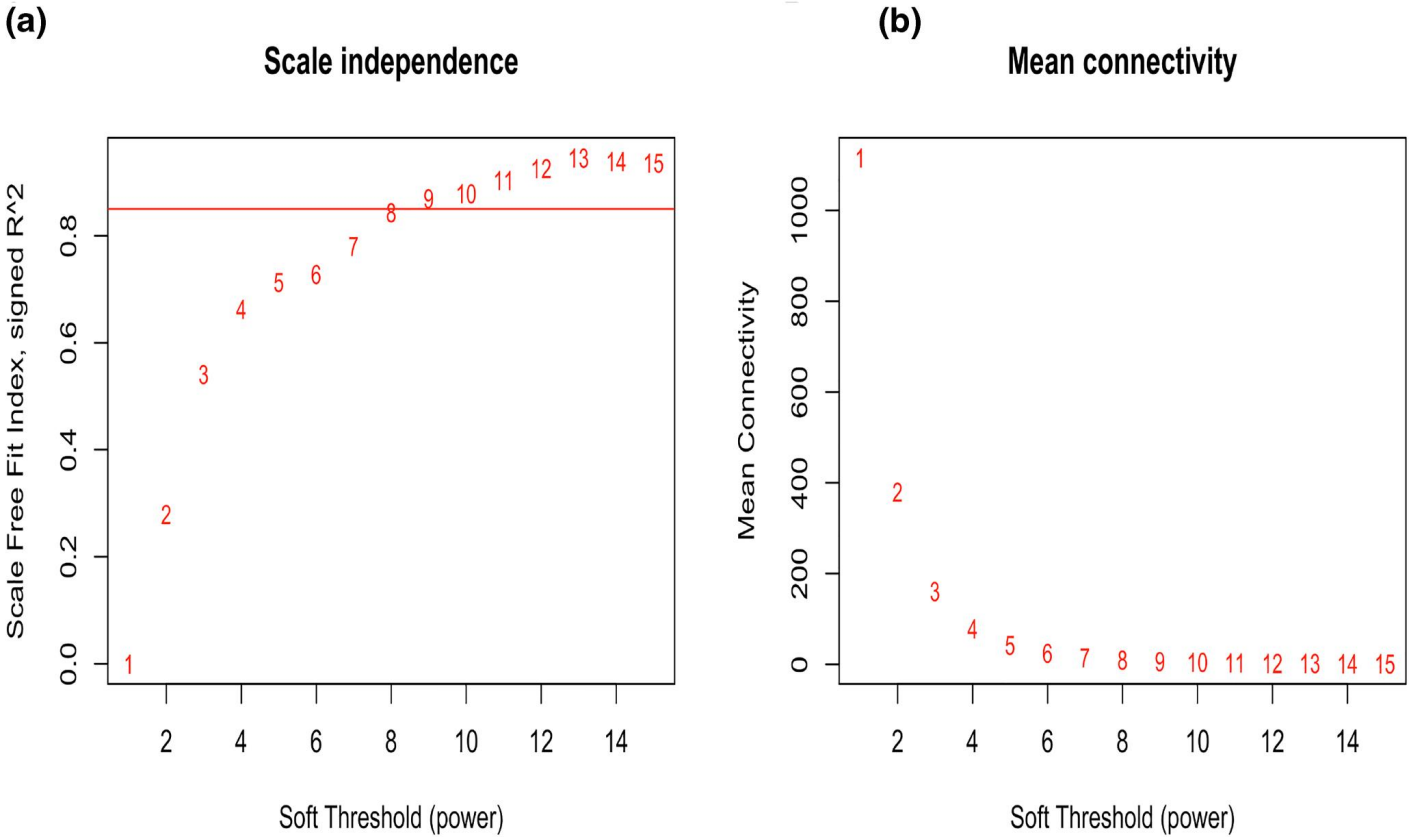
Analysis of soft‐thresholding values (β). (a) Calculation of scale‐free fit index of each β value from 1 to 15. (b) Calculation of mean connectivity of each β from 1 to 15

**FIGURE 4:**
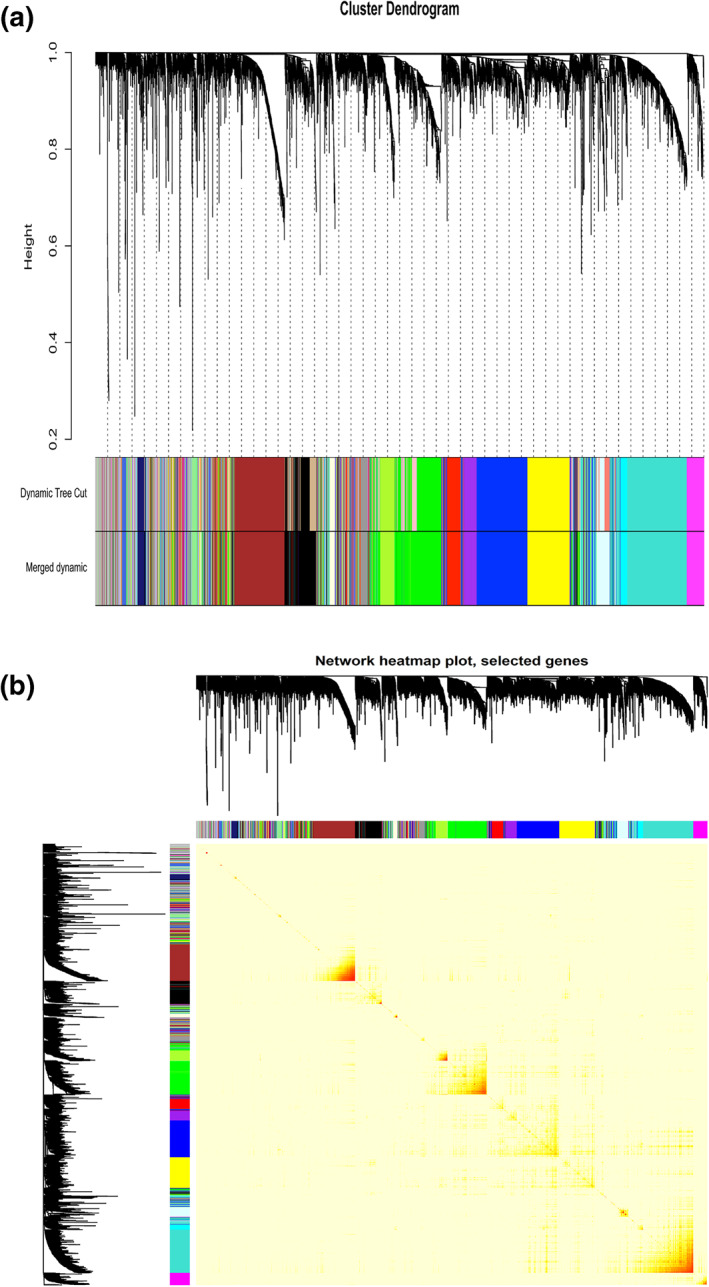
Division of co‐expression gene modules and an adjacency heatmap of genes. (a) Dendrogram of 5000 genes divided into 18 modules based on the TOM‐based dissimilarity measure. (b) Adjacency heatmap of 5000 genes analysed by weighted gene co‐expression network analysis (WGCNA). The correlation of pair‐wise genes was indicated by colour intensity

Based on the scale‐free fit index and mean connectivity, we selected nine as the most appropriate β value (Figures 3a and 3b). The expression matrix was then turned into a correlation matrix, which was subsequently translated into an adjacent matrix, and lastly into a TOM. Five thousand genes were divided into numerous modules, and after merging several similar modules, 18 modules were finally generated (Figures 4a and 4b). The green, green‐yellow, purple, yellow, royal blue, midnight blue, black, brown, magenta, light cyan, turquoise, light green, cyan, light yellow, grey 60, blue, red, and grey contained 609, 161, 168, 445, 38, 79, 386, 512, 175, 181, 718, 52, 85, 44, 66, 581, 249 and 451 genes, respectively. However, there were genes in the grey module that could not be split into any co‐expression modules.

### 3.4 Identification of significant clinically related hub module

**FIGURE 5:**
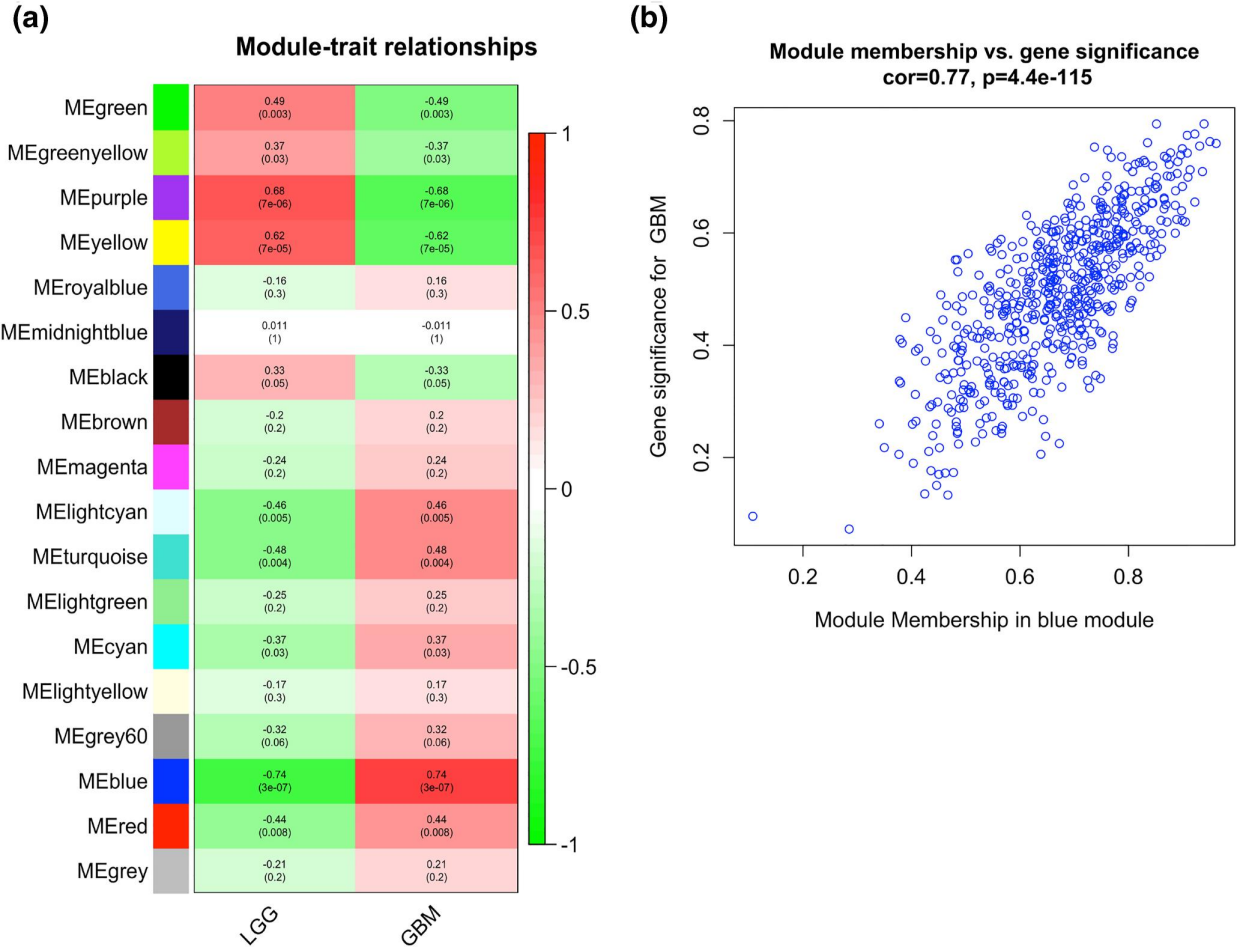
Identification of clinical‐related gene modules. (a) The heatmap of clinical trait‐module correlations. The intensity of correlation in each cell was indicated by colour intensity (red for positive and blue for negative correlation). The significances were shown in the cells. The blue module was identified as GBM‐related module. (b) Scatter plot exhibiting the correlation between gene significance (GS) and module membership (MM) in the blue module. The correlation coefficients and *p*‐value were shown at the top

We evaluated the correlation coefficients between clinical traits and gene modules. The results were exhibited as a heatmap (Figure 5a). We found that genes in the blue module were highly associated with the GBM trait with a high significance (*r* = 0.74, *p* = 3E‐07). Furthermore, we calculated the correlation of GS and MM in the GBM trait‐related blue module (cor = 0.77, *p* = 4.4E‐115) (Figure 5b). The selected GBM trait‐related module was the most appropriate module for subsequent analysis.

### 3.5 GO and KEGG enrichment analyses of the hub module

To investigate the biological function of genes in hub modules, we performed GO and KEGG enrichment analyses on the hub GBM modules and displayed the top 10 terms of GO and KEGG enrichment results. The results of GO and KEGG enrichment analyses are concluded in Table S2.

**FIGURE 6:**
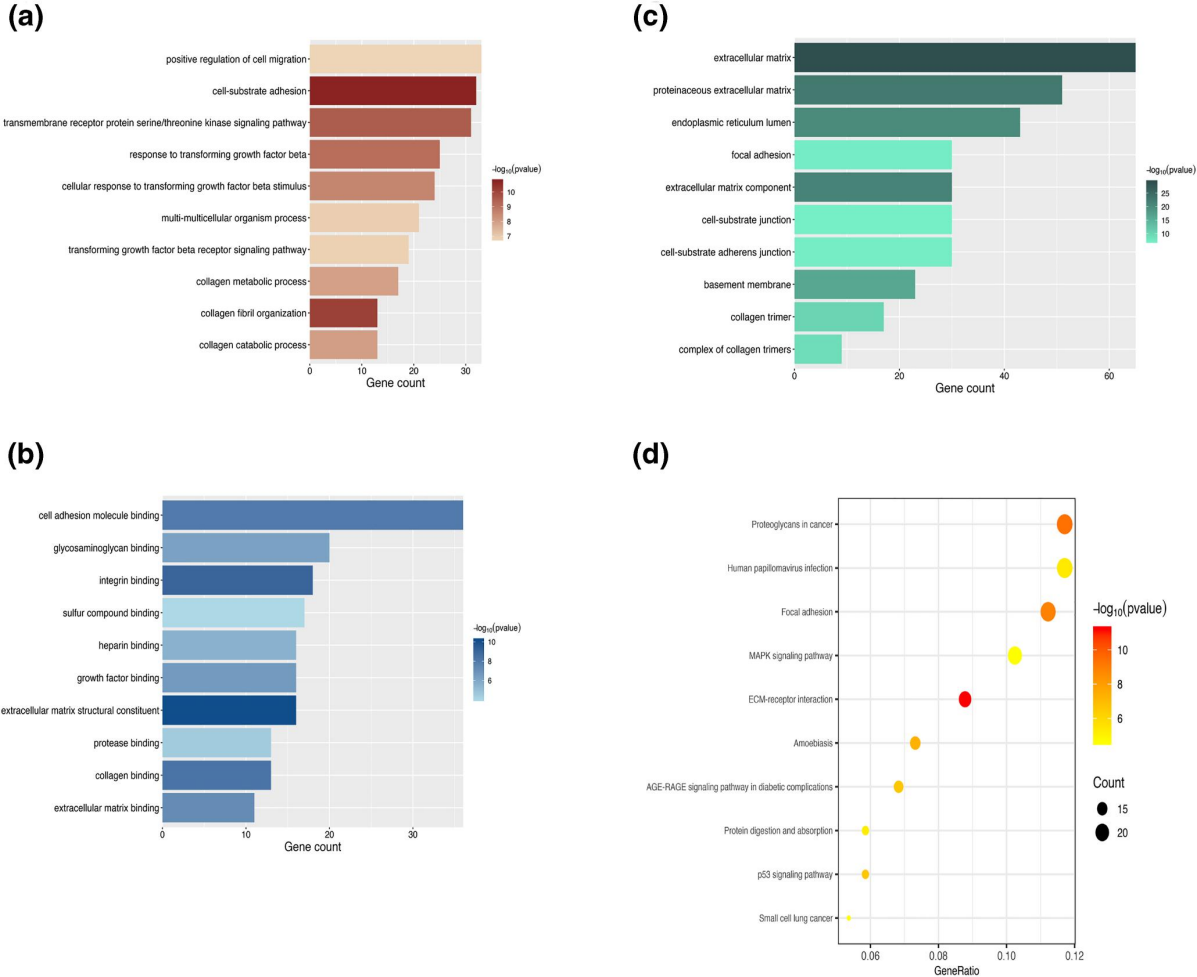
The gene ontology (GO) and Kyoto encyclopaedia of genes and genomes (KEGG) analyses of genes in the blue module. The length and colour of barplots in GO analyses represented the gene counts and statistical significances, respectively. The size and colour of dots in KEGG analyses represent the gene counts and statistical significance, respectively. (a) Top 10 GO‐BP terms. (b) Top 10 GO‐MF terms. (c) Top 10 GO‐CC terms. (d) Top 10 KEGG terms

We found that the top GO‐BP terms (Figure 6a) were associated with cell migration, transforming growth factor beta signalling pathway and collagen process, such as ‘positive regulation of cell migration’ (gene count = 33, *p* = 1.58E‐07), ‘transmembrane receptor protein serine/threonine kinase signalling pathway’ (gene count = 31, *p* = 2.96E‐10), and ‘collagen metabolic process’ (gene count = 13, *p* = 9.46E‐09). The top GO‐MF enriched terms (Figure 6b) were highly related with cell adhesion molecule, growth factor and extracellular matrix, such as ‘cell adhesion molecule binding’ (gene count = 36, *p* = 8.27E‐09), ‘growth factor binding” (gene count = 16, *p* = 2.27E‐07), and ‘extracellular matrix binding” (gene count = 11, *p* = 5.20E‐08). For GO‐CC, top terms were involved in the extracellular matrix and cell‐substrate junction (Figure 6c), such as ‘extracellular matrix ’ (gene count = 65, *p* = 6.06E‐30), ‘cell‐substrate junction’ (gene count = 30, *p* = 6.57E‐08), and ‘focal adhesion’ (gene count = 30, *p* = 4.20E‐08).

The findings of KEGG enrichment analysis were primarily regarding focal adhesion, which was similar to the conclusions of GO‐MF and GO‐CC, such as ‘focal adhesion’ (gene count = 23, *p* = 1.35E‐09), and some pathways that were reported to play an essential role in cancer progression, such as ‘p53 signalling pathway’ (gene count = 12, *p* = 2.59E‐07) and ‘MAPK signalling pathway’ (gene count = 21, *p* = 2.35E‐05).

### 3.6 Identification of hub gene

**FIGURE 7:**
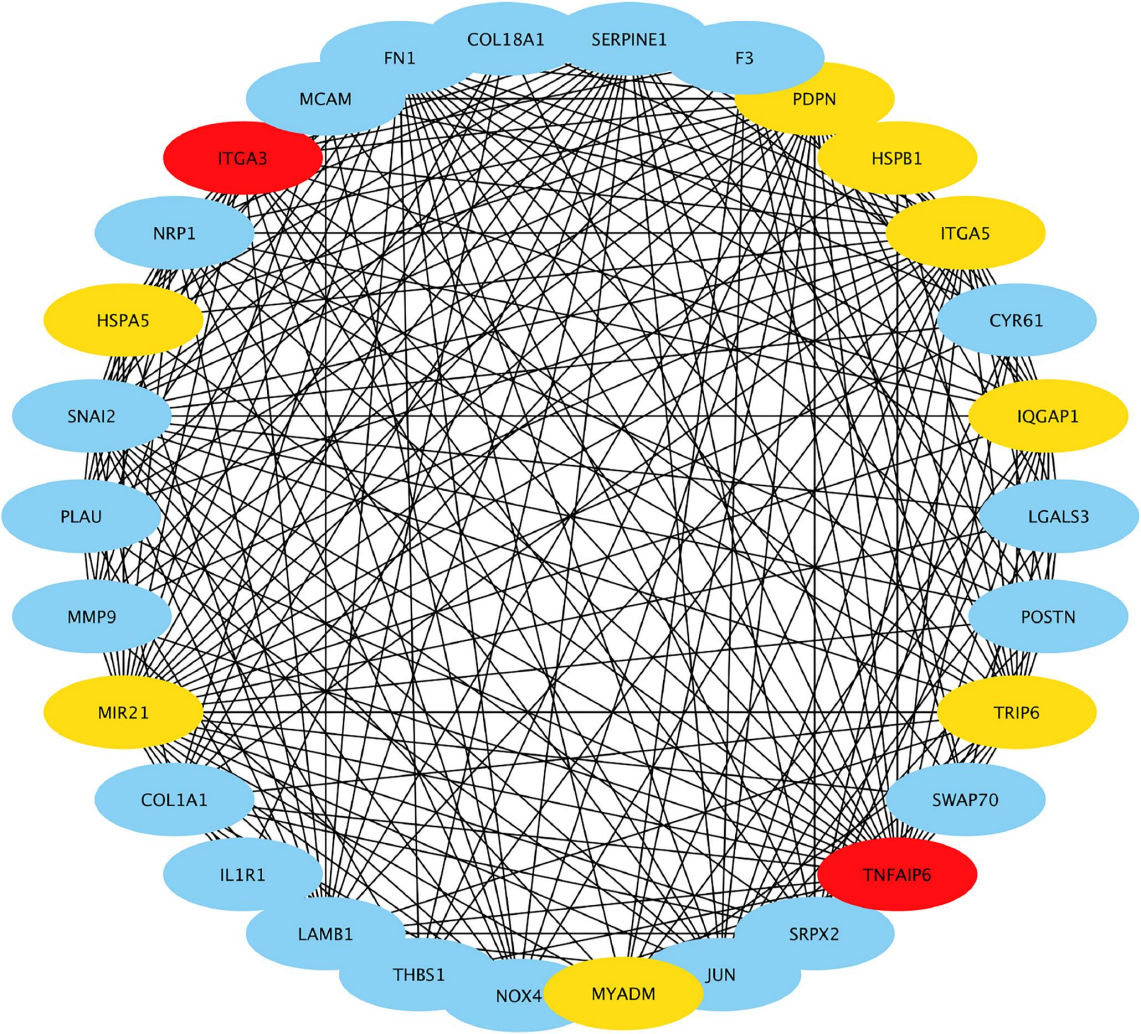
Identification of hub gene by maximal clique centrality (MCC) method. We calculated the MCC value of genes in the GO‐BP term ‘positive regulation of cell migration’. We displayed genes with top 10 MCC values in red and yellow, and red meant bigger MCC value

We extracted genes from the top GO‐BP term of ‘positive regulation of cell migration’ and used them to construct a new co‐expression sub‐network of these genes. Then, using the MCC approach in cytoHubba, we computed the centrality of the genes in the network. A high MCC score indicates a heightened connection with other genes and a high significance in the network. We identified the top 10 genes, including TNFAIP6, ITGA3, PDPN, HSPB1, ITGA5, IQGAP1, TRIP6, MYADM, MIR21, and HSPA5 in the network in Figure 7. Finally, we chose TNFAIP6 as our hub gene because it had the highest MCC value.

### 3.7 Validation of hub genes

**FIGURE 8:**
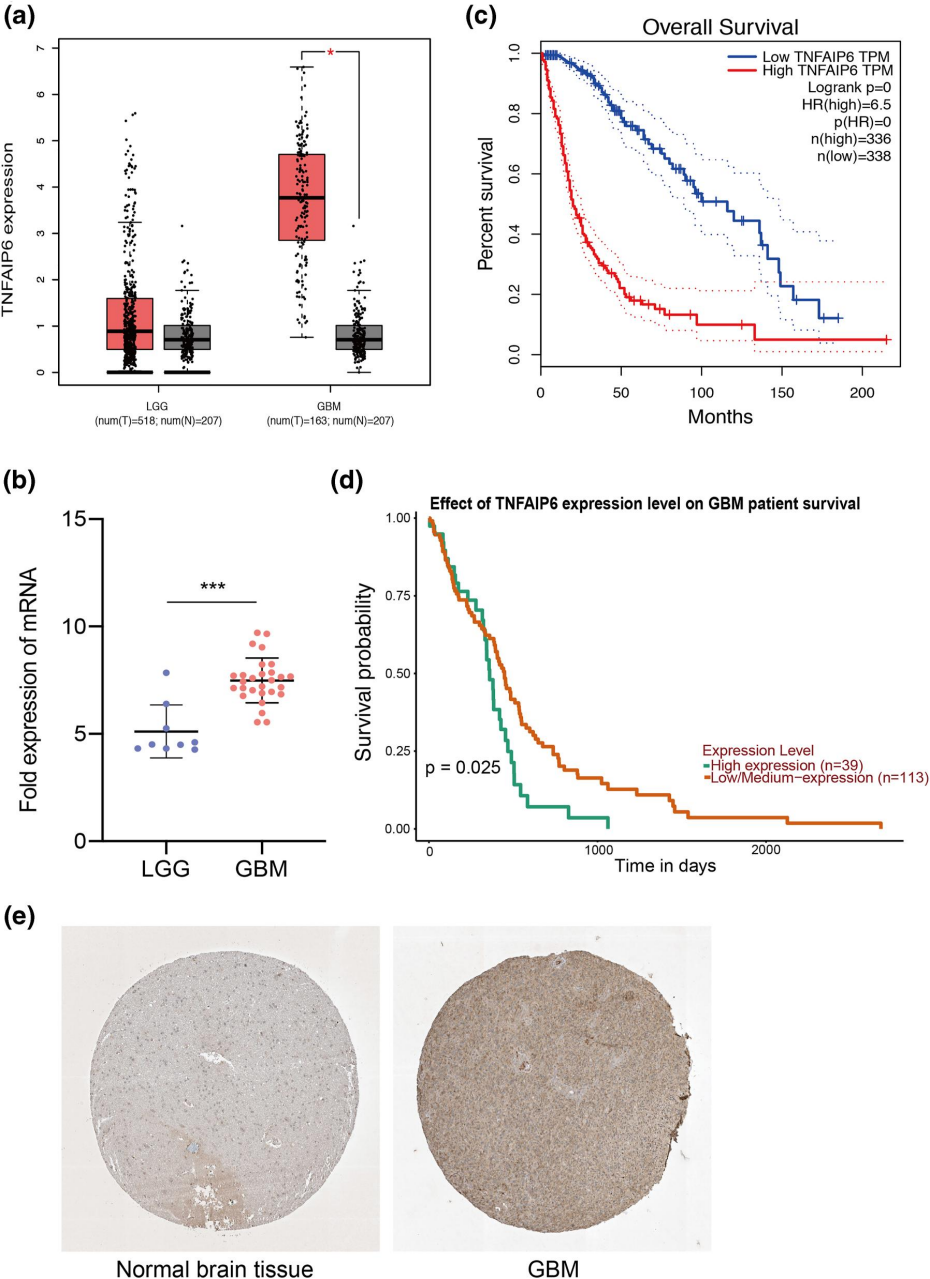
Validation of hub gene. (a) The expression level of hub gene TNFAIP6 in normal and patients of low‐grade glioma (LGG) and glioblastoma multiforme (GBM), respectively. Red box plots represented LGG and GBM patients, respectively and grey box plots represented normal people. (b) The expression level of TNFAIP6 in dataset of GSE43289. (c) Overall survival analysis on TNFAIP6 with gene expression profiling interactive analysis (GEPIA). Red line meant high expression level of TNFAIP6 while grey line meant low expression level. ***p < 0.001, LGG versus GBM

We used the online programme GEPIA to identify the relative expression level in LGG and GBM samples to testify to the function of the hub gene in the progression of GBM. We discovered that the expression of TNFAIP6 was equivalent in LGG samples compared to normal samples but increased dramatically in GBM samples compared to normal samples (Figure 8a). Furthermore, the TNFAIP6 expression was higher in GBM samples than in LGG samples (Figure 8b), showing that the level of TNFAIP6 increased with the advancement of GBM. In addition, we validated that GBM patients with higher TNFAIP6 expression exhibited a much worse prognosis and lower overall survival rate by survival analysis (Figures 8c and 8d). We next used the HPA database to detect the protein levels of TNFAIP6 and found that the expression level of TNFAIP6 was upregulated in the samples of patients with GBM (Figure 8e). On the other hand, we also validated the functions of another nine genes by the methods described above. We found that ITGA3, PDPN, HSPB1, ITGA5, IQGAP1, and TRIP6 were elevated in the GBM groups (Figures [Link], [Link]). Besides, high levels of ITGA3, PDPN, HSPB1, ITGA5, IQGAP1, TRIP6, MYADM, and HSPA5 were positively associated with a worse prognosis (Figure S4).

## 4 DISCUSSION

This study extracted the gene expression profile data from GSE43289 for WGCNA, covering 28 samples of glioblastoma patients and nine samples of LGG patients. Gene ontology enrichment analysis, KEGG signal pathway analysis, and survival analysis were used for subsequent analysis. The main findings of this study were as follows: 1. Analysis of the first 5000 genes with large variances identified 18 gene modules, of which 1 module (blue) was significantly positively correlated with the malignancy of the tumour. 2. Gene ontology analysis revealed that the genes of this module were mainly involved in cell migration, cell adhesion molecules, and extracellular matrix formation (ECM). In contrast, KEGG analysis revealed that these genes were related to proteoglycan, MAPK signalling pathway, and p53 signalling pathway. 3. TNFAIP6 was identified as a hub gene for glioblastoma that was significantly associated with the prognosis of tumour patients.

Our findings revealed that genes in the module of our interest were mainly involved in cell migration regulation. Tumour astrocyte cell migration is a complex and dynamic process, comprising at least three independent and highly coordinated biological processes [25]: [1] The cell adheres to several components of ECM and modifies ECM molecular composition; [2] the mobility of the cell itself is mainly achieved by modifying the field of integrin and ECM component to reorganise the actin cytoskeleton; [3] cell invasion primarily degrades matrix proteins via proteolytic enzymes secreted by tumours (including serine proteases, cathepsins, and matrix metalloproteinases (MMP)‐2, MMP‐9, and MMP‐14 (MT1‐MMP)). Glioma cells may create a suitable environment by manufacturing and accumulating modified ECM components to promote their migration. Several studies have discovered that some ECM components were upregulated in high‐grade glioma compared to LGG [26, 27]. Adhesion molecules with multiple cell surface receptor shape mediate interactions between glioma cells and ECM components. These findings corroborated our results. We can speculate that the key to glioblastoma's malignancy is much higher than that of LGG may be the difference in the expression of these genes involved in cell migration.

The results of KEGG analysis revealed that most of the genes in this module were primarily involved in proteoglycan, MAPK signalling pathway, and p53 signalling pathway; as we know, proteoglycans are an essential component of the brain ECM. Studies found that the expression of many proteoglycans fluctuated dramatically throughout the development of glioblastoma. For instance, perlecan is considerably upregulated in glioblastoma samples, and its high expression is linked to the poor prognosis in patients [28]. In GBM, the MAPK signalling pathway is primarily involved in cell proliferation, angiogenesis, and invasion. The current mainstream view believes that the activation of MAPK signalling pathway is required for GBM pathogenesis [29].

Furthermore, the activation of most MAPK pathways is positively correlated with the malignant phenotype of GBM. p53 pathway deregulation is highly prevalent in glioblastoma [30]. Deregulated p53 pathway components are associated with GBM invasion, migration, proliferation, apoptosis escape, and stemness of cancer cells, all of which are regulated by microRNAs and long non‐coding RNAs [31, 32, 33]. Their expression is linked to poor prognosis, highlighting their potential as a target for GBM therapy.

WGCNA is an effective bioinformatics tool to explore disease‐related hub genes. In previous investigations, most of the hub genes we discovered have been associated with glioblastoma. A study employing TCGA data, for example, found that high expression of the ITGA3 gene predicted a poor prognosis for glioblastoma [34]. Another study found that inhibition of HSPA5 could improve the efficacy of photothermal and radiotherapy for GBM [35]. Similarly, related reports confirmed that MIR21 [36], TRIP6 [37], ITGA5 [38], HSPB1 [39], PDPN [40], and IQGAP1 [41] all play a role in the pathogenesis or treatment of GBM. Based on previous reports, our study found that the expression levels of PDPN, HSPB1, ITGA5, IQGAP1, TRIP6, and ITGA3 were higher in GBM patients. Furthermore, high levels of these genes portended a worse prognosis. The previously mentioned results proved the reliability of our method. More notably, we discovered a new gene called TNFAIP6, which has strong co‐expression relationships with these hub genes. Previous research has not established a relationship between TNFAIP6 and GBM. Hence, the current study was the first to report the relationship between TNFAIP6 and GBM, which may provide a novel therapy target for the GBM.

TNFAIP6 is localised on the human 2q23.3 chromosome, also known as TNF‐stimulated gene‐6 [42], and encodes a secreted protein with anti‐inflammatory and tissue‐protective effects. TNFAIP6 usually is not expressed but is upregulated in various cells when exposed to inflammatory mediators and growth factors [43, 44]. TNFAIP6 expression is inhibited by anti‐inflammatory cytokines [43]. TSG‐6 (TNFAIP6 secreted protein) is a glycoprotein with a molecular weight of only 35–38 kDa, released by secreted granules of neutrophils, mast cells, and macrophages. It is combined with various ECM components such as glycosaminoglycans, proteoglycan core proteins, and other matrix components. They primarily serve to stabilise or reshape ECM [45]. Furthermore, TNFAIP6 has been found to have therapeutic effects in a variety of disease models, such as atherosclerosis [46], acute pancreatitis [47], type‐1 diabetes [48], and acute lung injury [49].

TNFAIP6 is also expressed in the brain and spinal cord. A basic experiment [50] discovered that TNFAIP6 was present in mature CD44+/GFAP+rat astrocytes but not during the CNS development. This experiment confirmed that TNFAIP6 was associated with astrocyte maturation, as TNFAIP6‐deficient mice have fewer astrocytes in the neocortex and hippocampus. TNFAIP6 was also significantly upregulated in spinal cord injury and might be a key component of glial scars bound to a matrix rich in hyaluronic acid. TNFAIP6 has also been linked to traumatic brain injury in other research. It can improve memory and depression‐like behaviour and increase the number of newborn neurons [51]. However, TNFAIP6 expression in brain tumours has yet to be investigated. This study found that TNFAIP6 plays a vital role in the occurrence and development of glioblastoma. The potential mechanism may be connected to the maturation of astrocytes. TNFAIP6 overexpression may result in malignant transformation of normal astrocytes. To establish the involvement of TNFAIP6 in glioblastoma, more basic experiments and sequencing data with a larger sample size are needed. This study has various limitations that must be addressed. First, the sample size of this investigation is relatively small, and no repeatability verification of independent datasets was undertaken. We recruited GBM patients from our brain centre to expand our study samples, and our findings will be validated in a future study. Second, only the clinical traits of tumour conditions were excavated and analysed, and no additional clinical traits were included. Finally, we speculate a new target for GBM, but subsequent basic experimental verification remains required. We will explore the function of TNFAIP6 in developing GBM in vivo and in vitro.

## 5 CONCLUSION

We identify TNFAIP6 as the hub gene in GBM progression by a series of bioinformatics studies with WGCNA as the core, and its high expression implies a poor prognosis for the patients. This discovery opens new avenues for understanding disease mechanisms and identifying novel therapeutic targets.

## CONFLICT OF INTEREST

There are no conflicts of interest to declare.

## Supporting information

Figure S1

Figure S2

Figure S3

Figure S4

Table S1

Table S2

## Data Availability

The datasets used and/or analysed in the current study are available from the corresponding author on reasonable request.
