# Supplementary figures and images for: Identification of TNFAIP6 as a hub gene associated with the progression of glioblastoma by weighted gene co‐expression network analysis
Source: IET Syst Biol. 2022 Jun 29;16(5):145–56. doi: 10.1049/syb2.12046 (PMC9469790; doi:10.1049/syb2.12046)

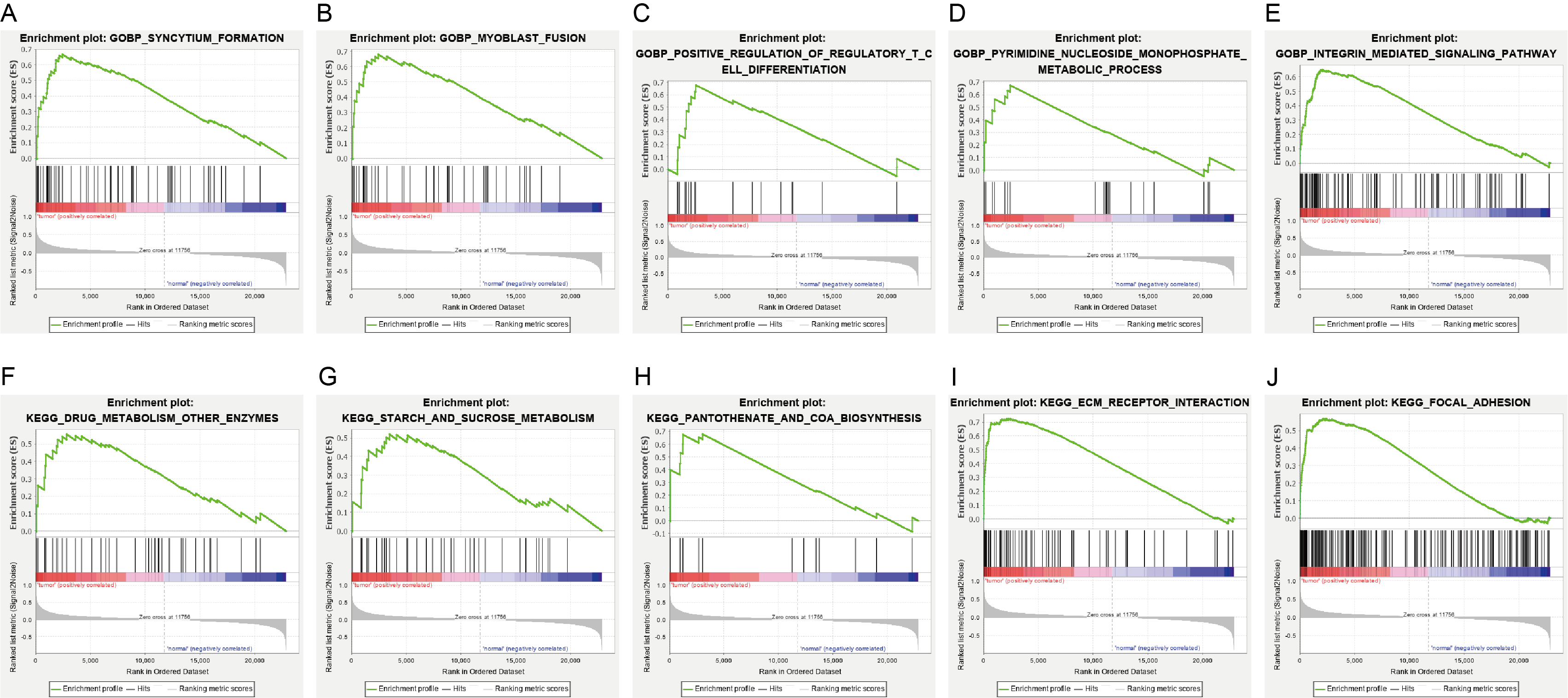

Supplement: Supplementary file 1 — Figure S1 [file SYB2-16-145-s006.png]

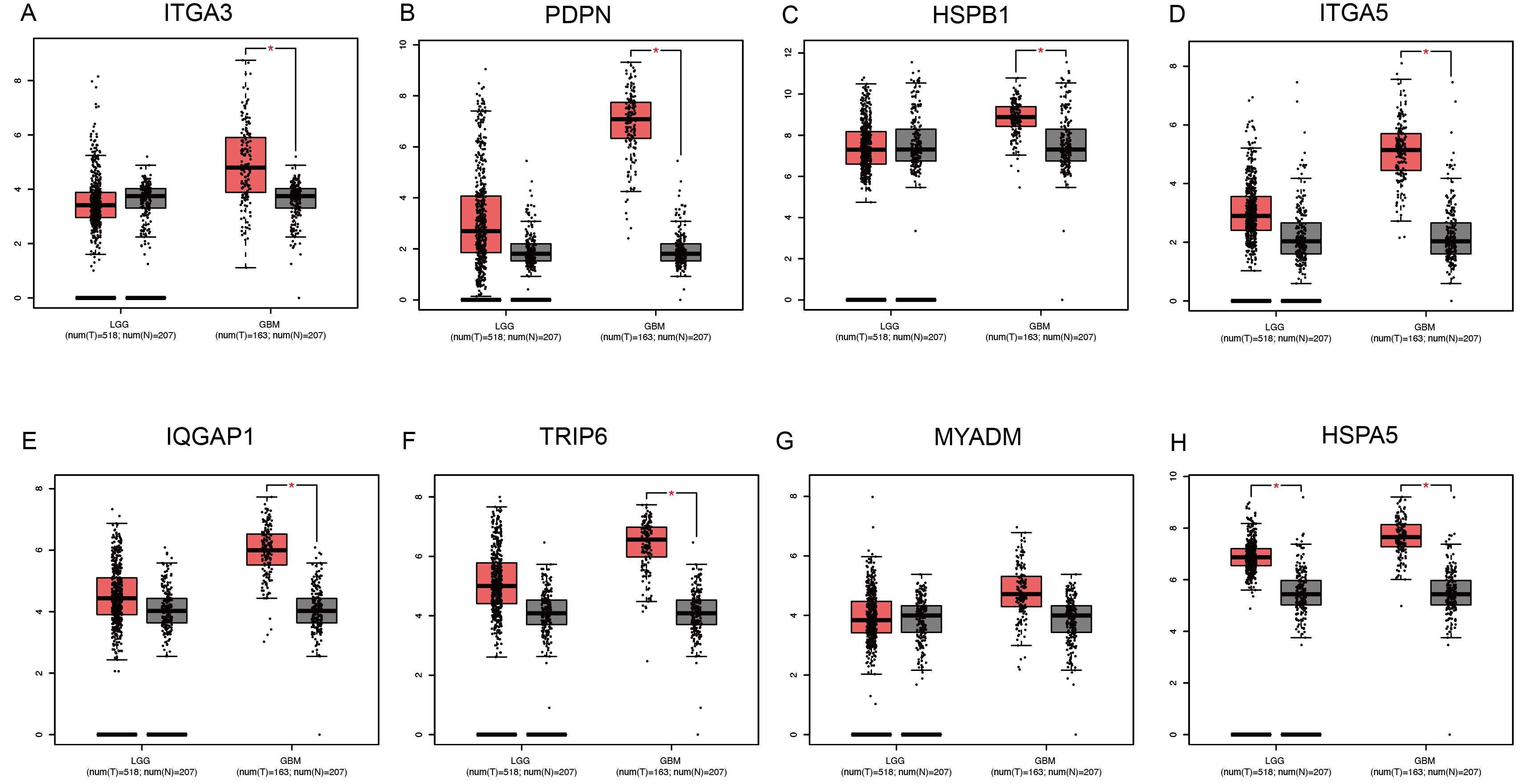

Supplement: Supplementary file 2 — Figure S2 [file SYB2-16-145-s002.png]

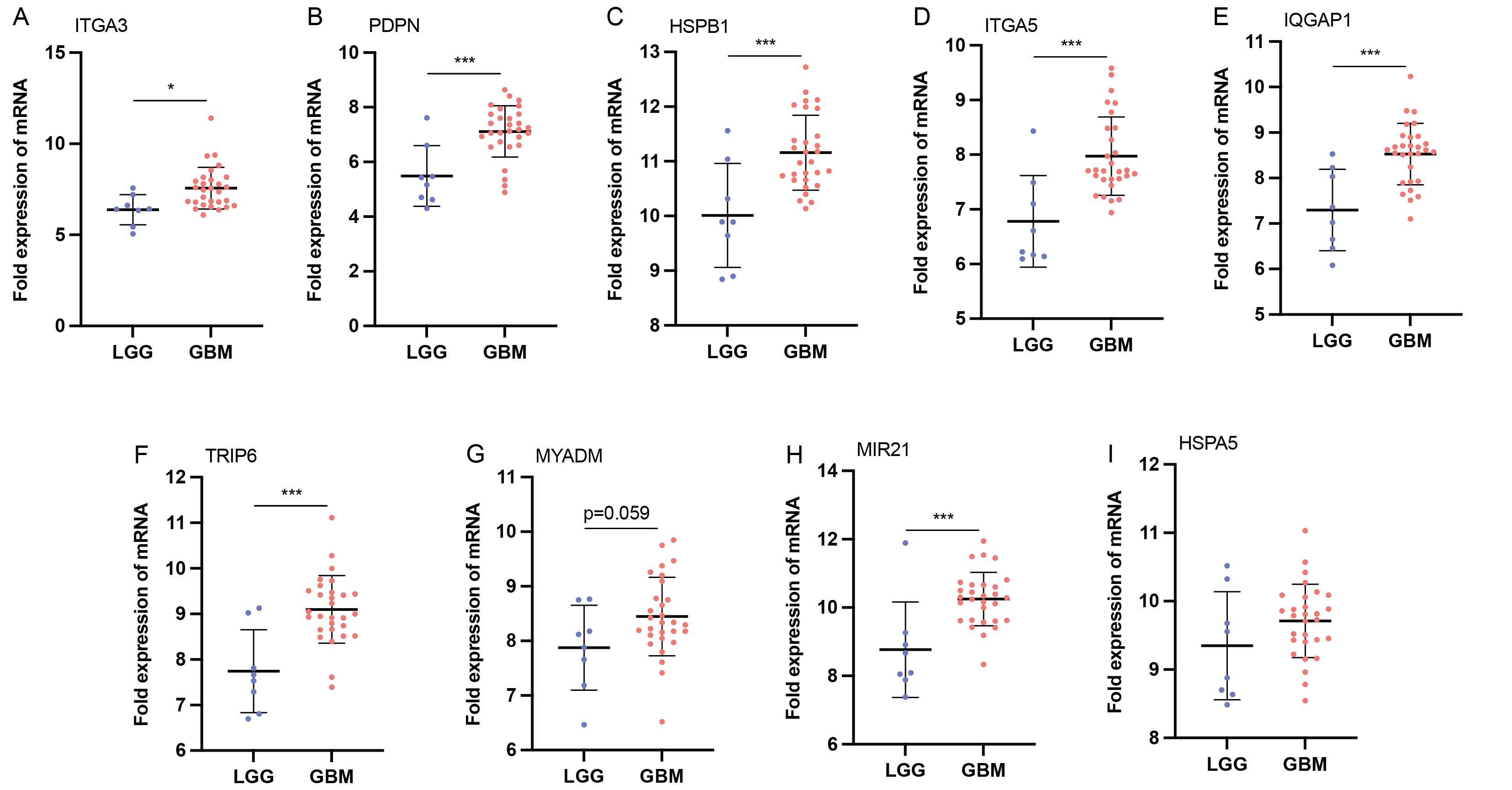

Supplement: Supplementary file 3 — Figure S3 [file SYB2-16-145-s004.png]

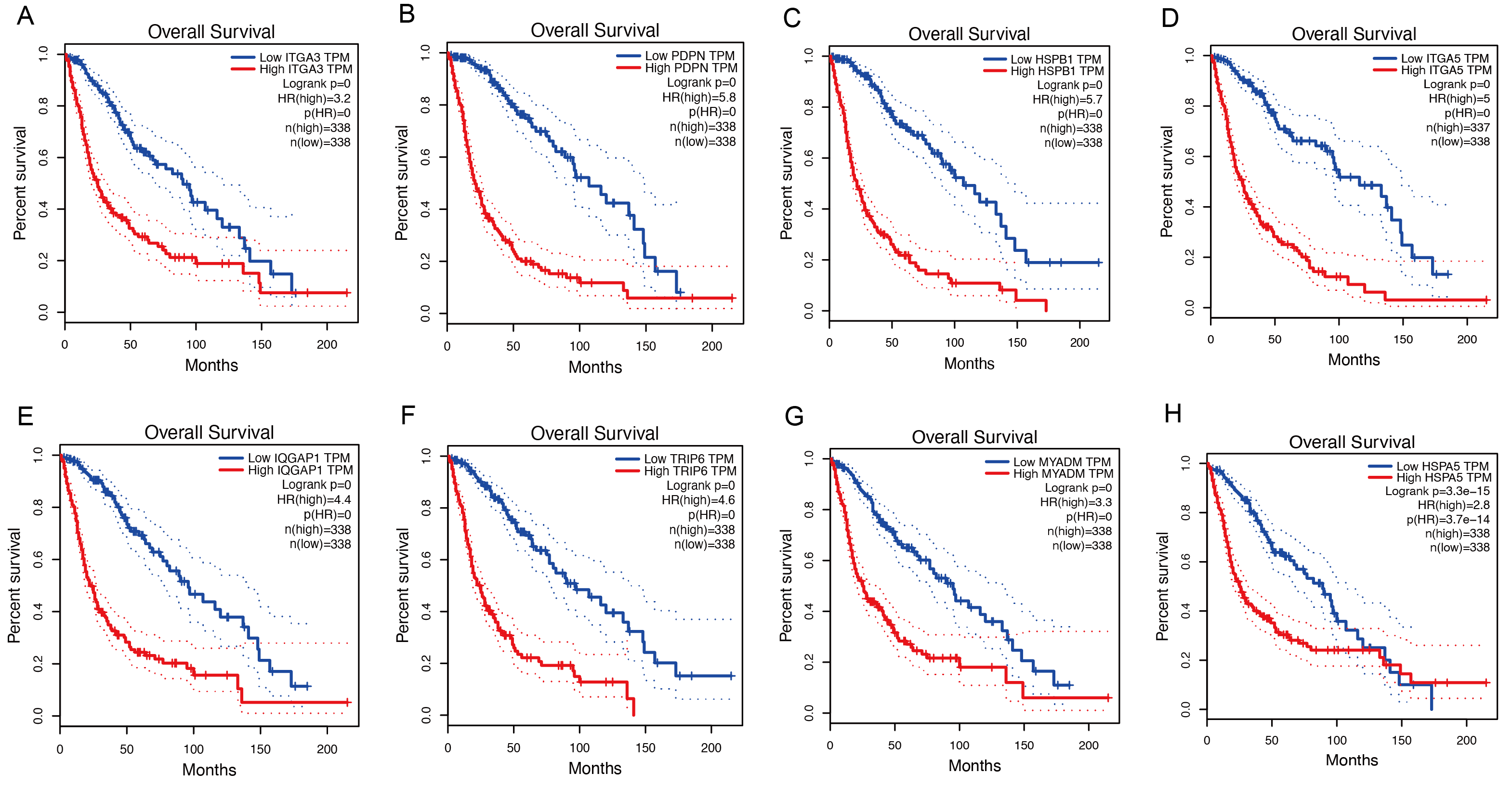

Supplement: Supplementary file 4 — Figure S4 [file SYB2-16-145-s003.png]
